# CaRMS at 50: Making the match for medical education

**DOI:** 10.36834/cmej.69786

**Published:** 2020-07-15

**Authors:** John Gallinger, Michel Ouellette, Eric Peters, Lisa Turriff

**Affiliations:** 1 Canadian Resident Matching Service (CaRMS), Ontario, Canada; 2 Paediatric Anesthesia, The Hospital for Sick Children, Ontario, Canada

## Abstract

Entry into postgraduate medical training in Canada is facilitated through a national application and matching system which establishes matches between applicants and training programs based on each party’s stated preferences.

Health human resource planning in Canada involves many factors, influences, and decisions. The complexity of the system is due, in part, to the fact that much of the decision making is dispersed among provincial, territorial, regional, and federal jurisdictions, making a collaborative national approach a challenge. The national postgraduate application and matching system is one of the few aspects of the health human resources continuum that is truly pan-Canadian.

This article examines the evolution of the application and matching system over the past half century, the values that underpin it, and CaRMS' role in the process.

## Abstract (fr)

L’entrée dans la formation médicale postdoctorale au Canada est facilitée par un système national de demande et de jumelage qui établit des correspondances entre les candidats et les programmes de formation en fonction des préférences déclarées par chaque partie.

La planification des ressources humaines en santé au Canada implique de nombreux facteurs, influences et décisions. La complexité du système est due, en partie, au fait qu'une grande partie du processus décisionnel est réparti entre les compétences provinciales, territoriales, régionales et fédérales, ce qui rend difficile une approche nationale collaborative. Le système national de demande postdoctorale et de jumelage est l'un des rares aspects du continuum des ressources humaines en santé qui est véritablement pancanadien.

Cet article examine l'évolution du système d'application et de jumelage au cours du dernier demi-siècle, les valeurs qui le sous-tendent et le rôle du CaRMS dans le processus.

Entry into postgraduate medical training in Canada is facilitated through a national application and matching system which establishes matches between applicants and training programs based on each party’s stated preferences. The Canadian system is similar to the process followed in the United States. The Canadian and American residency matches follow a central clearinghouse model featuring preferences expressed through rank order lists from both applicants and training programs.^1^ A match algorithm compares applicant and program rank order lists and matches applicants to programs based on both parties’ stated preferences.

While the Canadian and American models are markedly similar, residency selection methods in other countries vary. Spain, France, and Portugal, for example, employ a national exam and base admission to residency exclusively on academic criteria,^2^ while in other European centres the process is handled locally by medical disciplines with applicants “admitted to the available spots based on an assessment of their skills, enthusiasm, and so on.”^2^

Dr. Inge Schabort, International medical graduate coordinator at McMaster University’s Department of Family Medicine, observes:

In my country of origin the usual practice would be to be approached by the Chair of a department to let you know you are one of the ‘chosen ones’ for a residency position - do you want to apply - and that is how I received my first residency position. It was only when I came to Canada and had to apply for a residency position again that I became aware of an independent organization that conducted the residency match at arm’s length with clear, transparent criteria specifying the ranking and matching processes. This was very different from my previous experience and my introduction to the Canadian culture of fairness and transparency.^3^

Health human resource planning in Canada involves many factors, influences and decisions. The complexity of the system is due, in part, to the fact that much of the decision making is dispersed among provincial, territorial, regional and federal jurisdictions, making a collaborative national approach a challenge.^4^ One of the few aspects of the health human resources continuum that is truly pan-Canadian is the national postgraduate application and matching system.

A good match produces better outcomes both for physicians and the patients they serve. That’s why it’s so important to have a fair and objective match process that advances the interests of both medical learners and training programs. The Canadian Resident Matching Service (CaRMS) has been integral to this system’s evolution over the past 50 years and is working with all stakeholders to ensure it adapts successfully to the needs of tomorrow.

## Why the match?

The Canadian match for postgraduate medical training was first established by the Canadian Association of Medical Students in 1969, in response to what was then a hectic, decentralized application and selection process.

Offers came in piecemeal, on no predictable schedule, and applicants often had to consider one offer at a time without knowing their prospects at other programs. An applicant receiving their first offer from a second- or third-choice program had to decide whether to take that offer or decline it in hopes that another would arrive from their first choice; and if they gambled and lost, they could be left stranded. Because training programs were also scrambling to secure their desired candidates, time-limited offers were common, heightening the anxiety for applicants. The decision-making process for offers and acceptances was not clear, and applicants would sometimes not feel safe to express their true preferences.^5^ Dr. Ian Bowmer, President of the Royal College of Physicians and Surgeons of Canada and former faculty dean recalls his own experience during this time: “it was very opaque, inequitable and somewhat chaotic applying and getting a residency position when I went through it.”^3^

Many faculties also had frustrations with the pre-match system, as they had to seek out applicants through informal channels earlier and earlier in order to secure their most desired candidates. And when a first-choice applicant rejected an offer, it was often too late for a program to secure its next preferred candidate.

A national, coordinated match was seen by the medical students as one way to improve the process for everyone involved. But the students had challenges with both finances and securing the cooperation and participation of hospitals, who ran the training programs at the time.

## Why CaRMS?

In 1970, key stakeholders in the medical education community – including the Association of Canadian Medical Colleges (ACMC) (now renamed as the Association of Faculties of Medicine of Canada – AFMC) representing the faculties, as well as physician associations, learner organizations and medical licencing and regulating authorities – banded together with the Canadian Association of Medical Students to help fully realize the idea of an open, fair and transparent match for postgraduate medical education.

These stakeholders determined that a key ingredient to the success of the match, in addition to a broad base of community support, was a dedicated organization to oversee the process. This is when CaRMS’ earliest incarnation, the Canadian Intern Matching Service (CIMS), was created under the umbrella of the ACMC for the purpose of administering the match.

It became evident in relatively short order that CIMS would be better able to perform its function as an impartial steward of the match if it were an independent organization. This led to the emergence of CIMS as an independent, not-for-profit entity in 1982.^6^ According to Dr. Andrew Padmos, former CEO of the Royal College of Physicians and Surgeons of Canada and Canadian Association of Interns and Residents representative on the CIMS Advisory Board from 1974 to 1976: “Without a direct stake in individual outcomes, CIMS was uniquely suited to run a fair and unbiased residency application, selection and match process, and to be a platform where policies could be implemented provincially and nationally.”^3^

The CaRMS of today is built on these strong foundations. Its governance model draws on a broad base of stakeholders in the medical education community to ensure the representation of all perspectives in its policies and procedures, while still maintaining its independence.^7^

This independence remains a crucial aspect of CaRMS’ role in matching more than 4,000 medical students and residents to training positions each year. As Dr. Emily Stewart, President of Resident Doctors of Canada, notes, “an independent body administering the process is an integral part of the Match.”^3^

## The value of the match

The match’s raison d'être is as compelling today as when it was first introduced. In the words of former CaRMS Executive Director Sandra Banner, “the policies governing the match were built around safety, fairness and equity, which remain core values today and have been strengthened by the system’s evolution over the years.”^3^

Dr. Armand Aalamian, Postgraduate Dean at McGill, highlights transparency, clear rules and consequences for violating those rules as integral to an effective application, selection and match process.^3^ While compliance and accountability were uneven in the beginning—with programs occasionally offering positions outside of the established process, failing to adhere to common timelines and pressing for informal commitment from applicants—evolution over the years has brought a strong tradition of adherence to policies and processes. The CaRMS Violations Review Committee today provides crucial oversight and accountability, addressing the rare exceptions that arise. As former Canadian Federation of Medical Students (CFMS) president Dr. Henry Annan observes, “there is an inherent hierarchy between learners and faculty, and having an independent body overseeing the process helps ensure that it functions as intended and that those hierarchies do not result in conflicts of interest.”^3^

The match recognizes the importance of choice for both applicants and programs, while ensuring medical schools can host the number, mix and distribution of positions that best allow them to meet their social accountability mandate to the population as determined by provincial health human resources plans. Since the needs of the health care system dictate the opportunities available, applicants may need to consider a range of options—but no applicant will end up in a training program in which they do not want to train, and no program will receive a trainee it considers unacceptable. “Participants place their trust in the process,” says Fédération des médecins résidents du Québec president Dr. Christian Campagna, “because it makes it safe for them to pursue what they want without politics and geographic limitations.”^3^

In fact, the Match Algorithm prioritizes applicants’ choices to give them the best chance of matching to their preferred programs. The algorithm traverses each applicant’s rank order list downwards, from most preferred program to least preferred, until the first program to which the applicant can be tentatively matched is reached, or until the applicant’s list of choices is exhausted. Each program accepts applicants upwards on its rank order list, continually removing less preferred tentative matches in favour of more preferred applicants, until the program is matched to the most preferred applicants who wish to be matched to the program. At the end of the process, each applicant has either been matched to their most preferred choice possible, or all choices submitted by the applicant have been exhausted and they have not been matched.^8^ “This prioritization of the student’s choice is an essential element of the way the match works,” says Dalhousie Family Medicine Department Head David Gass. “It puts them in the driver’s seat on their career path.”^3^

## How the application and selection system has evolved

The CaRMS match of today, with its national scope, was a far-off vision when CIMS was created. In the match’s early days, not all programs chose to participate. The match’s national scope was a gradual achievement over many years as greater numbers of programs participated and higher proportions of positions were offered and filled through the match. It wasn’t until 2005, after several years of discussion, that Québec’s French-language medical schools joined to make the match a truly national institution. As the 2000s progressed, the community’s appetite for applying the benefits of the match beyond R-1 resulted in the introduction of the three residency subspecialty matches CaRMS continues to offer today.

One of the most transformational milestones in the evolution of the match was the introduction of a centralized and standardized application package and process in 1988. Developing a central application necessitated a great deal of deliberation about what information and documents would be included, as well as the processes surrounding it. Before the central application was introduced, there was a hard deadline for application submission, but not a hard start. Applicants could send applications to programs at different times, resulting in inequalities around the amount of time applications could be reviewed and allowing the possibility of first-come, first-served bias. The central application changed all of this, creating a level playing field by bringing in a match-wide schedule with common deadlines and standards.

Other key changes along the way helped shape the match, and CaRMS, into its current form. In 1993, rotating internships following medical school were replaced with the current multi-discipline residency model.^9^ The move from rotating internships to thirty separate discipline-specific entry routes, which completely reimagined what medical training looked like, was the result of much discussion and debate. CaRMS, of course, adapted to serve this new direction in post-graduate training and then changed its name to the Canadian Resident Matching Service, or CaRMS, as it is known today. 1993 was also the first year the match was run in two iterations. The second iteration was introduced as an opportunity for unmatched graduates to consider and apply for positions that were not filled in the initial run of the match.

The match process underwent another fundamental change in 2002 when CaRMS launched its online application process. This was made possible by CaRMS’ understanding of the need to keep pace with emerging technology to serve its clients, which led to investments in an in-house IT department to focus on the development of specialized enabling technology.

Today, CaRMS’ online platform continues to evolve with changes in technology and community needs. Sharing information between applicants and programs for decision making purposes has taken on sharper focus through recent enhancements to support Best Practices in Application and Selection (BPAS)^10^ with a focus on transparency and fairness.

Since the match’s inception, there has been a burgeoning appetite for data around its outcomes. CIMS, and then CaRMS, has produced detailed match data reports for every match since its first in 1971.^11^ As both community needs and technology have advanced, the nature of the data produced has grown in both quantity and quality—moving toward increasingly sophisticated analytics and data visualizations that can help stakeholders understand not only the outcomes but also the underlying factors and influences to aid in decision making.

Some of this historical data can shed light on the way the match and its participants have changed. In the 1971 match, the first administered by CaRMS, 578 students participated (including 68 international medical graduates), vying for a total of 809 positions.^12^ Fast forward to 2019 and the R-1 match includes 4,746 applicants (of which 1,725 were international medical graduates)^13^ and 3,346 positions.^14^

**Figure 1:**
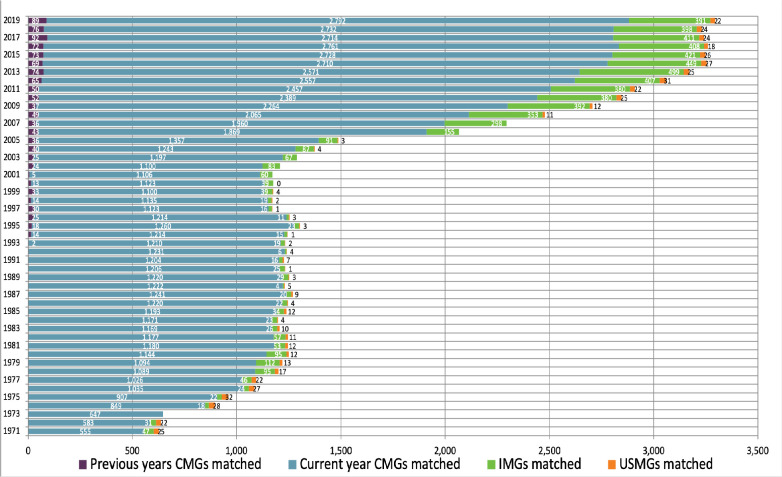
Matched applicants as part of the R-1 match 1971-2019

**Figure 2:**
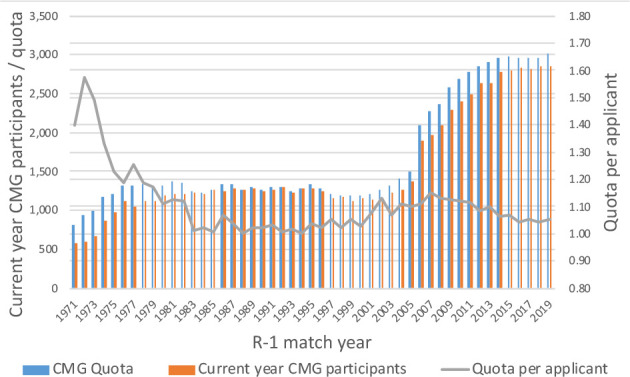
Current year CMG R-1 participation and quota *Note: To ensure consistency of data over time, participant data includes current year CMG applicants only*.

**Figure 3:**
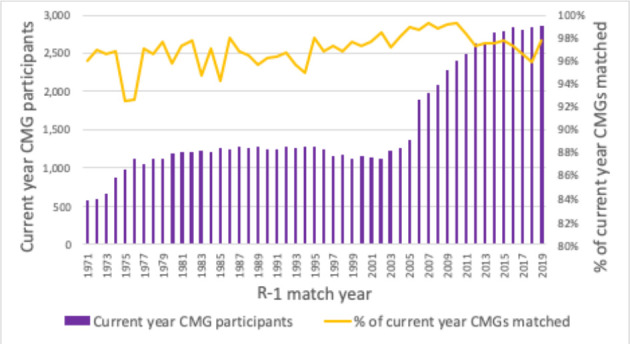
Current year CMG R-1 participation and match rate *Note: To ensure consistency of data over time, participant data includes current year CMG applicants only*.

**Figure 4:**
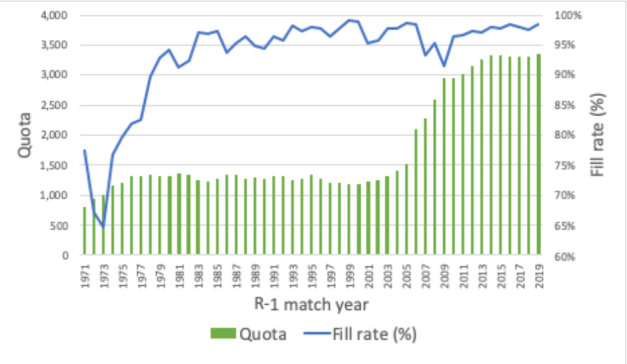
R-1 quota and position fill rate

**Figure 5:**
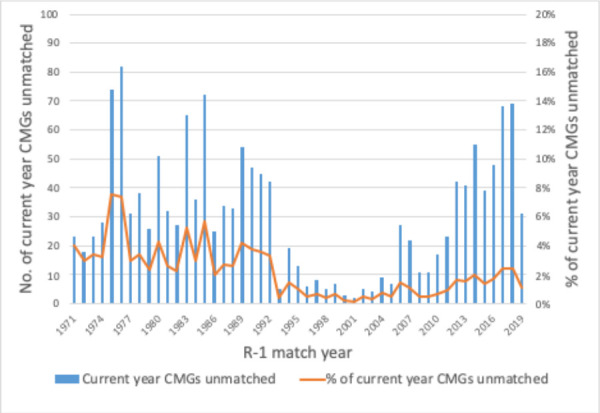
Unmatched current year CMGs in the R-1 match

The charts below, which synthesize data from historical CaRMS match reports, tell the story of a system that has grown and evolved over time. Striking changes can be seen over the first 10 years as the system matured and stabilized. Impacts from the introduction of the second iteration and elimination of the rotating internship can be seen in match rates, unmatched CMG rates and fill rates. (See Figures 1-5).

While international medical graduates have been a part of the match from the beginning, there has been significant growth in this cohort over the years. Since 2008, more than 300 international graduates have entered residency training annually through the match.

The most important measure of the match, of course, is its effectiveness. The two primary gauges of system efficacy—match rates and position fill rates—paint a picture of a system that is responsive to the basic inputs. Critical relationships can be seen between the number of positions and participants and the outcomes of applicant match rates and quota fill rates. As one might expect, generally speaking when the ratio of positions to participants is high, match rates are high and fill rates drop. The reverse is also true, as you can see in the longitudinal data in this article.

There are of course many other influential factors. Two such factors which impact outcomes are policy changes concerning eligibility, and shifts in discipline supply and demand based on position availability and applicant preference.

## Looking toward the future

The overall effectiveness of the match at the system level is clear. When looking at the match rates in Figure 3, the average yearly match rate for the past seven years is 97 per cent. This statistic is complemented by the fact that 99.4 percent of Canadian medical graduates match in Canada within three years.^15^

Continuous improvement has always been a critical feature of the application, selection and match system. It needs to continue to evolve to keep pace with changing societal needs, and the emergence of new technology opens up new opportunities to rethink how things are done. As Postgraduate Dean at McGill Dr. Aalamian remarks, “the system cannot continue to be effective without ongoing evaluation and quality improvement of processes. It requires an ability to adapt based on feedback and the needs of stakeholders.”^3^

Former CFMS president Dr. Annan notes that, “historically, the medical community has been slow to change, but perhaps the application, selection and match system can actually be a catalyst for change.”^3^ As steward of an important part of the system, CaRMS is working with stakeholders throughout the medical education sphere to address challenges as they arise and ensure the system continues working at its best for everyone.

One of the most visible challenges the medical education community has been contending with in recent years is the issue of unmatched Canadian medical graduates. While there have always been Canadian graduates who do not match in their first year of participation as seen in the table above, there is no doubt that the prospect of being unmatched can add stress to applicants as they complete the application process—and going unmatched can be a very difficult outcome to deal with for the applicants affected. Accordingly, the situation has been the focus of a great deal of thought and action, including in the AFMC’s 2018 report “Reducing the number of unmatched Canadian Medical Graduates: A way forward” which points to the complexity of addressing the issue when “the primary stakeholders involved in, and impacted by, the increasing number of unmatched CMGs each have different priorities.”^16^

Considering that applicants spend a great deal of time on their applications and on critical decisions, more information could help reduce the anxiety and uncertainty inherent in the process. Recent changes to program descriptions that incorporate Best Practices in Application and Selection (BPAS)^10^ are an example of efforts to better inform applicants. CaRMS will continue working with clients to explore solutions—technological and otherwise—to make the process and decision making easier and more effective.

Congestion, to borrow a term used by Alvin E. Roth, co-winner of the 2012 Nobel Prize in Economic Science for his research into market design and game theory, is another challenge within the current system.^17^ A key feature of the Canadian system is the freedom for applicants to pursue every available opportunity; at the same time, increases in the number of applications each applicant submits are resulting in system congestion and administrative burden for many programs. Tools to help programs more easily manage their processes are routinely being developed and fine-tuned.

In addition, other influences such as emerging technology, Competency Based Medical Education, the call for increased diversity in the medical education system, and the evolution of what are considered necessary skills, abilities and characteristics of physicians may impact the current system. In particular, the issue of supply and demand as manifested through the alignment – or lack thereof in some cases – between the provincial health human resources decisions that inform available positions and the discipline preferences of match applicants is an ongoing challenge.^18^ No doubt, these and other changes in the medical education continuum will impact the application, selection and matching system. Collectively, we will need to be nimble and respond appropriately and swiftly while at the same time ensuring we do not compromise a system that, in the last five decades, has matched more than 80,000 learners to their next career opportunity.

CaRMS remains committed to working with the medical education community to ensure the system evolves to meet their future needs—whatever that tomorrow may bring.
